# Ammonia removal from simulated fish farms by metal organic framework ingrained by egg shell and fish bones

**DOI:** 10.1038/s41598-025-01827-0

**Published:** 2025-05-16

**Authors:** Ebtehal A. H. Gamal, Reda M. Abdelhameed, Hossam E. Emam, Hanan B. Ahmed

**Affiliations:** 1 Lakes & Fish Resources Protection & Development Agency, The 5th Settlement, New Cairo, 11835 Egypt; 2 Applied Organic Chemistry Department, Chemical Industries Research Institute, National Research Centre, Scopus affiliation ID 60014618, 33 EL Buhouth St, Dokki, Giza, 12622 Egypt; 3 Department of Pretreatment and Finishing of Cellulosic Fibers, Textile Research and Technology Institute, National Research Centre, Scopus affiliation ID 60014618, 33 EL Buhouth St, Dokki, Giza, 12622 Egypt; 4 Chemistry Department, Faculty of Science, Helwan University, Ain-Helwan, Cairo, 11795 Egypt

**Keywords:** Egg shell, Fish bones, Biogenic Ca-precursor, Ca-MOF, Ammonia removal, Fish farms, Adsorption, Pollution remediation, Materials science

## Abstract

The current work focuses on the efficient removal of ammonia by calcium-based metal organic framework. Whereas, for the first time, Ca based metal organic framework was synthesized using either fish bones (FB) or eggshell (ES) as biogenic wastes to act as calcium precursors for synthesis of Ca-BDC, abbreviated as Ca-BDC(FB) & Ca-BDC(ES), respectively, to be sequentially applicable for removal of ammonia. The collected data revealed that; FB showed to be more preferable rather than ES as calcium precursor for preparation of highly efficient Ca-BDC. The obtained Ca-BDC (ES) and Ca-BDC (FB) were shown with orthorhombic crystals, while smaller crystal size was observed in case of Ca-BDC (FB) which is reflected in larger surface area (721.38 m^2^/g) and in turn higher absorptivity for more efficient removal of ammonia. The adsorption of ammonia followed the pseudo-second ordered reaction and Langmuir isotherm, also R2 values of second ordered and Langmuir models were estimated to be 0.98 & 0.99 for Ca-BDC (ES) & Ca-BDC (FB), respectively. The evaluated maximum adsorption capacities (Q_m_) onto ES & Ca-BDC (ES) were 86.99 mg/g and 308.16 mg/g, respectively. The maximum capacities of ammonia onto FB & Ca-BDC (FB) were 184.28 mg/g and 616.11 mg/g, respectively. Whereas, Ca-BDC (FB) (721.38 m^2^/g) was shown with significantly wider surface area by factor of 1.3 compared to Ca-BDC (ES) (563.16 m^2^/g). Overall, the study provides a promising trend for in designing MOFs with appropriate central metals for capturing of NH_3_.

**Supplementary Information:**

The online version contains supplementary material available at 10.1038/s41598-025-01827-0.

## Introduction

Ammonia (NH_3_) as common pollutant in agricultural production is also known as a highly toxic gas. Also, ammonia has stimulating effects on the human eyes and upper airways, even in low concentrations. A high ammonia emission has brought extensive hidden dangerous effects to the surrounding environment and economy^1–3^. Therefore, environmental pollution with ammonia is known as a serious problem that could arouse consideration from the worldwide society. Moreover, the researching interests in discharging of ammonia pollution have been extensively considered among relevant departments of the government and academia^4–6^. It is well known that fertilizer manufacturing, refrigeration industry, fossil fuel combustion, coke manufacturing and poultry & animal husbandry can emit a large quantity of ammonia^7,8^. On the other hand, most of aquatic invertebrates and fishes act in producing ammonia (NH_3_ and NH_4_^+^) as the main nitrogen-based wastes that is formed within the protein catabolic process. To a lower degree, fishes also can produce urea (CO(NH_2_)_2_) and some of amino acids. Also some types of fishes can excrete proteins directly as body mucus^9–11^. Meanwhile, fish can excrete NH_3_ and NH_4_^+^ from its skin and gills or discharge it in the burst with the stored urine in urinary bladder^10,12,13^.

In a previous report about the rainbow trout, other potential nitrogen-containing products such as tri-methyl amine, tri-methyl amine oxide, uric acid, nitrites, and nitrates were not estimated; whereas creatine was estimated with ≤ 1.4% of total nitrogen-excretion^9^. In teleost fish, the plasma total ammonia concentration was normally varied between 0.05 and 1 mmol/L^14^, however, the plasma ammonia level reaches 2 mmol/L, and the sensitive species such as the arctic char can exhibit flaccid paralysis and other observable negative effects. Moreover, the fish can degrade the proteins and synthesizes the ammonia in the mitochondria and cytosol of their cell (hepatocytes, astrocytes, and others) and in the guts, where the microbial cells, especially −ve gram bacteria and yeast, can participate in the digestion or fermentation of proteins^14,15,16^.

Although the physiological role for ammonia, but fishes can produce extensively more than the required amounts, thus the excess ammonia must be eliminated to maintain the homeostasis. Any conditions that prevent the excretion of ammonia, especially the high environmental ammonia, will cause the endogenous ammonia accumulation within the fish cells, resulting in toxic effects to the homeostatic mechanisms that ammonia normally acts in its regulation. Whereas, the overabundance of ammonia leads to flatten the trans-membrane components, reduce the production of ATP, swelling of cells (especially brain astrocytes and hepatocytes), acidosis, generation of reactive oxygen species (ROS), and oxidative damaging^17–19^.These dramatic effects can manifest as the fish gasping for the air and exhibiting the increased cholesterol levels, plasma cortisol, and glucose, that promotes the depletion of energy storage, lethargy, and poor growing rate/reproductive action, and disturbance of the immune functions^18,19^.

Adsorption is superiorly investigated for the removal of different wastes via the application of solid materials as adsorbent for elimination of different pollutants. Meanwhile, the adsorbents were mainly selected to be able to attract and surface-bonded to the pollutants. According to literature, different types of adsorbents were applied for the removal of various pollutants such as “silica gel”^20–24^, “activated alumina”^21,25,26^, “biochar”^27^, “activated carbon”^25,28,29^, “metal–organic frameworks”^30–33^, “ion-exchange resins”, “modified polymers”^34,35^ and “zeolites”^36–38^ in addition to “solid acids and modified silica–alumina”^39,40^.

Zeolite molecular sieves, activated carbon, and metal oxides, are previously applied as conventional ammonia adsorbents for solving the ammonia pollution. However, they are disadvantageous with low selectivity and low capacity for ammonia removal^41–43^. However, MOFs (metal–organic frameworks), as a characteristic porous material, are advantageous with large specific surface area, high porosity, ordered porous structure and modifiable composition^44–47^. MOFs are widely applied in molecular recognition, separation, gas storage and catalysis, owing to their merits^48–50^. Moreover, MOFs were hypothesized as a promising material for ammonia removal^51–55^. Among the different types of MOFs such as ZIF-8, Cu-BTC, UiO and MIL series were widely exploited for ammonia removal, whereas, ammonia adsorption capacity of ZIF-8 was low^56^. However, Cu-BTC, is reported to have a high ammonia uptake affinity^57^ as it could strongly interact with ammonia due to the presence of sensitive metal sites. Moreover, MOFs with the immobilization of different functional groups were also applied for ammonia removal. It was reported that^58^, the immobilization of biphenyl and bi-pyridine groups into UiO-67 and UiO-bpydc could result in drastically various ammonia adsorption action attributing to the bi-pyridine moiety can promote the flexibility to the framework without significant alteration of the pore volume. Moreover, it is also reported that MIL-100 and MIL-101 exhibit a high affinity for ammonia uptake of 8 and 10 mmol/g, respectively, and the adsorption capacity of ammonia could be enhanced via the modification of the amino functional groups. Therefore, it could be decided that; all of the MOFs is superiorly characterized with excellent stabilization and recyclability for ammonia removal^59^. Although some common types of MOFs were reported for the purpose of ammonia adsorption^60,61^, but according to our knowledge no reports were considered with Ca-based MOF for such purpose.

Among the different species of metal organic frameworks that are reported for adsorption applications, MOFs that is composed of biocompatible metallic center like calcium are extensively considered in relation to the first-row of transition metals or the lanthanide analogues^62–68^. Whereas, the advantageous characters of calcium such the cost effectiveness, the low toxicity and bio-abundant are reflected in extensive attraction of the attention for studying its affinity for preparation of Ca-based MOFs in potential industrial purposes. As previously mentioned that, most of the conventional techniques for ammonia removal are disadvantageous with low selectivity and low capacity, while, MOFs are superiorly characterized with excellent stabilization and recyclability. Herein, the current approach uniquely represents a comparable study between fish bones (FB) and egg shells (ES) as biogenic wastes to act as calcium precursors for synthesis of Ca-BDC via solvo-thermal technique, abbreviated as Ca-BDC(FB) & Ca-BDC(ES), respectively. Whereas, BDC is for first is in the current study was prepared from biowastes in order to obey the green chemistry concept of waste treat waste. The as-prepared Ca-BDC was subsequently studied for their affinity in ammonia removal from fish farms. The as-synthesized Ca-BDC was well-characterized via the scanning electron microscope, energy dispersive X-ray, X-ray diffraction, BET and infrared spectra. The adsorption kinetic key factors, adsorption isotherm and regeneration were also studied.

## Experimental

### Chemicals

Terephthalic acid (benzene 1,4-dicarboxylic acid, BDC, C_8_H_6_O_4_, 98%), acetic acid (glacial, CH_3_COOH, ACS reagent, ≥ 99.7%), *N*,*N*-dimethyl formamide (DMF, C_3_H_7_NO, ACS, reagent, ≥ 99.8%), ethyl alcohol (CH_3_CH_2_OH, ≥ 95%), were all obtained from Sigma-Aldrich and applied without purification. Fish bones and eggshell of chicken were collected from the wastes of restaurant in Giza-Egypt.

#### Synthesis of Ca-acetate

According to literature^69^, the collected eggshells (ES) and fish bones (FB) as natural based wastes of calcium were cleaned up by washing with tap water for ten minutes followed by distilled H_2_O, and then kept in atmospheric air to discharge the non-preferable odor. The cleaned eggshells and fish bones were separately dried in oven at 100 °C for 12 h to be sequentially grinded, to obtain Ca_3_(PO_4_)_2_ and CaCO_3_ from fish bones (FB) and eggshells (ES), respectively. Afterward, acetic acid (40%) was added with weight ratio of 1:2 (acetic acid: calcium wastes). The mixture was agitated at ambient conditions for 60 min until the full evolution of carbon dioxide gas, to be dried at ambient conditions for successive production of calcium acetate which identified as the pale white powder.

#### Synthesis of Ca-BDC

To produce Ca-BDC via the solvothermal procedure according to literature^64^, 0.042 g of terephthalic acid and 0.0316 g of the prepared Ca-acetate were mixed in 4 mL mixture of deionized water/ethanol (3:1 mL) and then the mixture was heated at 25 °C for 20 min. The reaction mixture was put in a reactor and placed in oven at 90 °C. After one day, the precipitate was filtrated by Whatman filter papers, washed by ethanol and then dried in atmospheric conditions. The yield of prepared Ca-BDC (ES) powder from egg shells was 42.32 mg and the yield of prepared Ca-BDC (FB) powder from fish bones was 44.1 mg. The elemental analysis (Table S1, supplementary file) of Ca-BDC (ES) showed 43.2% C, 2.75% H and 18.1% Ca^2+^, while, the elemental analysis of Ca-BDC (FP) showed 40.1% C, 3.36% H and 16.68% Ca^2+^.

### Analysis and characterization

The topographical features of the synthesized Ca-BDC (ES) and Ca-BDC (FB) were investigated using high-resolution scanning electron microscope “HRSEM, Quanta FEG 250 FEI, Netherland”. The powdered samples were added to a carbon gride to be well-analyzed with the microscope. The elemental analysis and the chemical composition were identified with the energy dispersive X-ray (EDX) analyzer. X-ray diffraction (XRD) analysis for the synthesized Ca-BDC (ES) and Ca-BDC (FB) were collected with “Philips X’Pert MPD diffractometer (Kα X-radiation at 45 kV, 40 mA and λ = 1.5406 Å)” at room temperature and copper was used as monochromatic. The spectral maps of the diffracto-grams were estimated at the diffraction angles (2θ) of 3°–50°. The synthesized Ca-BDC (ES) and Ca-BDC (FB) were analyzed via Fourier transformation infrared (FTIR) spectroscopy “JASCO FT/IR-4700 spectrophotometer; Japan” in order to identify their chemical composition and functionality. Transmission (T%) spectral maps were collected in the range of “500–4000 cm^–1^, while the spectral data were smoothed with 15 points and 64 repetitive scans with the interval of 2.0 cm^− 1^”. The surface properties (surface are; BET, pore size and pore volume) of the synthesized Ca-BDC were all measured by the NOVA touch 4 LX Quantachrome version 1.21. Degassing of samples was carried out at 100 ◦C under vacuum, while the N_2_ adsorption was done at 77 K.

### Adsorption of ammonia

The synthesized Ca-BDC (ES) and Ca-BDC (FB) were applied in the removal of ammonia from aqueous solution. The concentration of ammonia was determined by using the ammonia-Nessler method^70^. Nessler Reagent (K_2_HgI_4_) is used to detect ammonia and it was prepared by addition of potassium iodide (7 g) and mercury iodide (10 g) to sodium hydroxide (16 g) in water. It was reacted with a solution of ammonia producing dirty brown precipitate. The adsorption of ammonia was tested using the 100 mL synthetic ammonia solution (50 mg/L) with 0.5 g of Ca-BDC powder. The mixtures were stirred by using multi-position magnetic stirrer at 250 rpm at room temperature (25 °C ± 2). The adsorption experiments were run at ambient room temperature of 27 °C. The content of ammonia was measured at different contact time (20–160 min) until the equilibrium reached. The supernatant was filtered by Whatman filter paper (0.45 μm) and the residual ammonium concentration in the form of ammonium chloride were detected using Nessler method. A 10 mL of the filtrate was taken out and then 2 drops of potassium sodium tartrate and 1 mL of Nessler reagent were added. The colour of ammonia contain samples was changed related to the ammonia content and subsequently, the ammonia concentration in the samples were measured using Shimadzu UV-2401 spectrophotometry. The experiment was repeated twice and the average values were presented. According to literature [….], the ammonia removal performance was calculated using the following Eq. (1):

1 $${\text{R}}\,=\,{C_f} - {C_p}/{C_f} \times {\text{1}}00$$

where *C*_*f*_ and *C*_*p*_ are the concentration of ammonia (mg/L) in the initial solution and after adsorption, respectively.

## Results and discussion

### Characterization of Ca-BDC

**Fig. 1:**
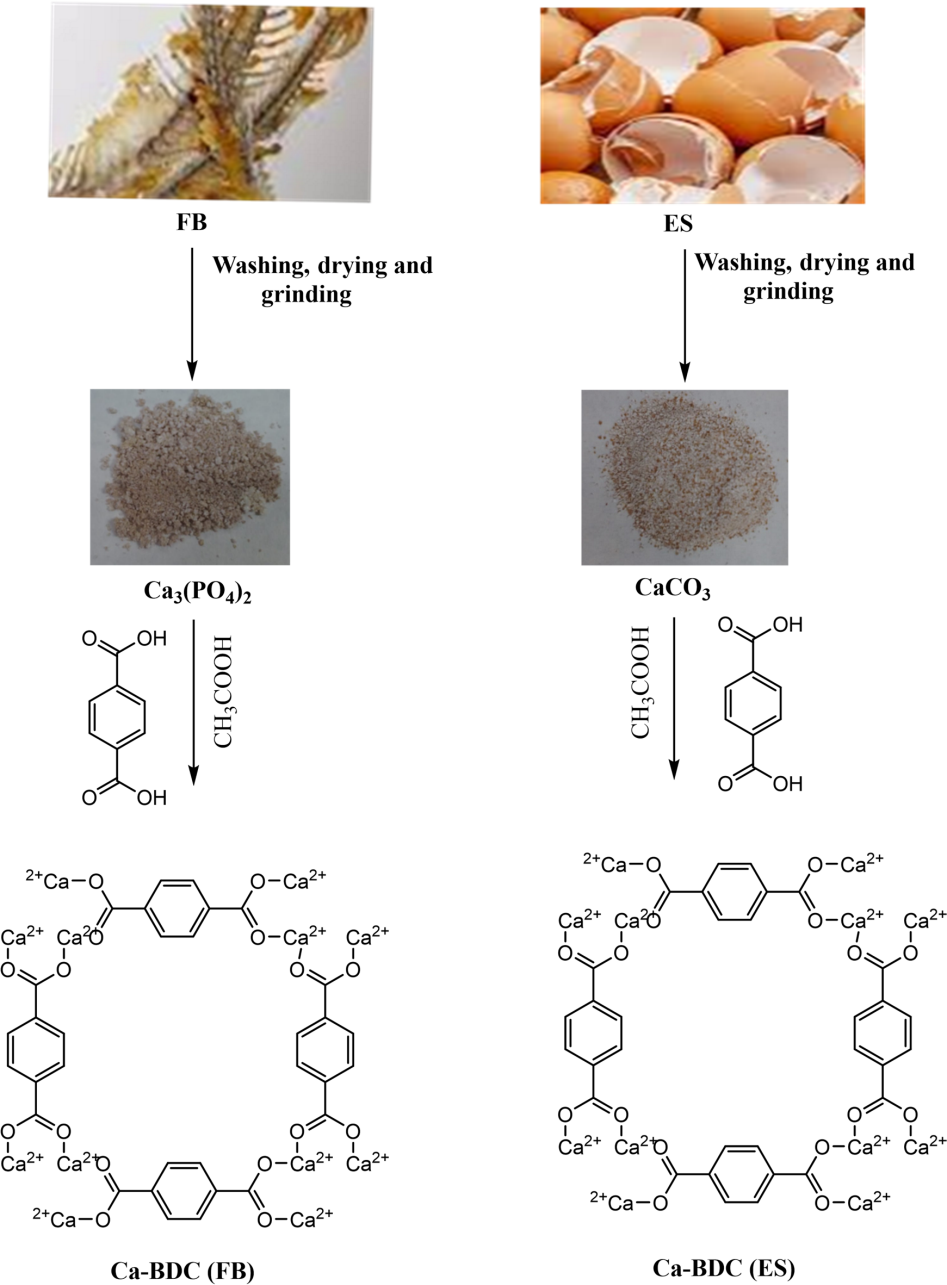
Preparation scheme of Ca-BDC from Egg shell (ES) and Fish bones (FB).

ES and FB as bio-wastes for calcium-source were used for synthesis of Ca-BDC as schematic in Fig. 1. After drying the wastes, the calcium acetate was formed by solubilizing of waste in the acetic acid. Ca-BDC was then obtained by interaction of calcium acetate with terphthalic acid through an electrophilic substitution reaction. The hydrogen of carboxylate in terphthalic acid is replaced by Ca^2+^ and covalent bonds formed between oxygen and Ca to give Ca-BDC, beside the formation of coordination bonding between COO- in BDC and Ca centers. The by-product of acetic acid was removed by washing process and the pure Ca-BDC (ES or FP) was obtained.

**Fig. 2:**
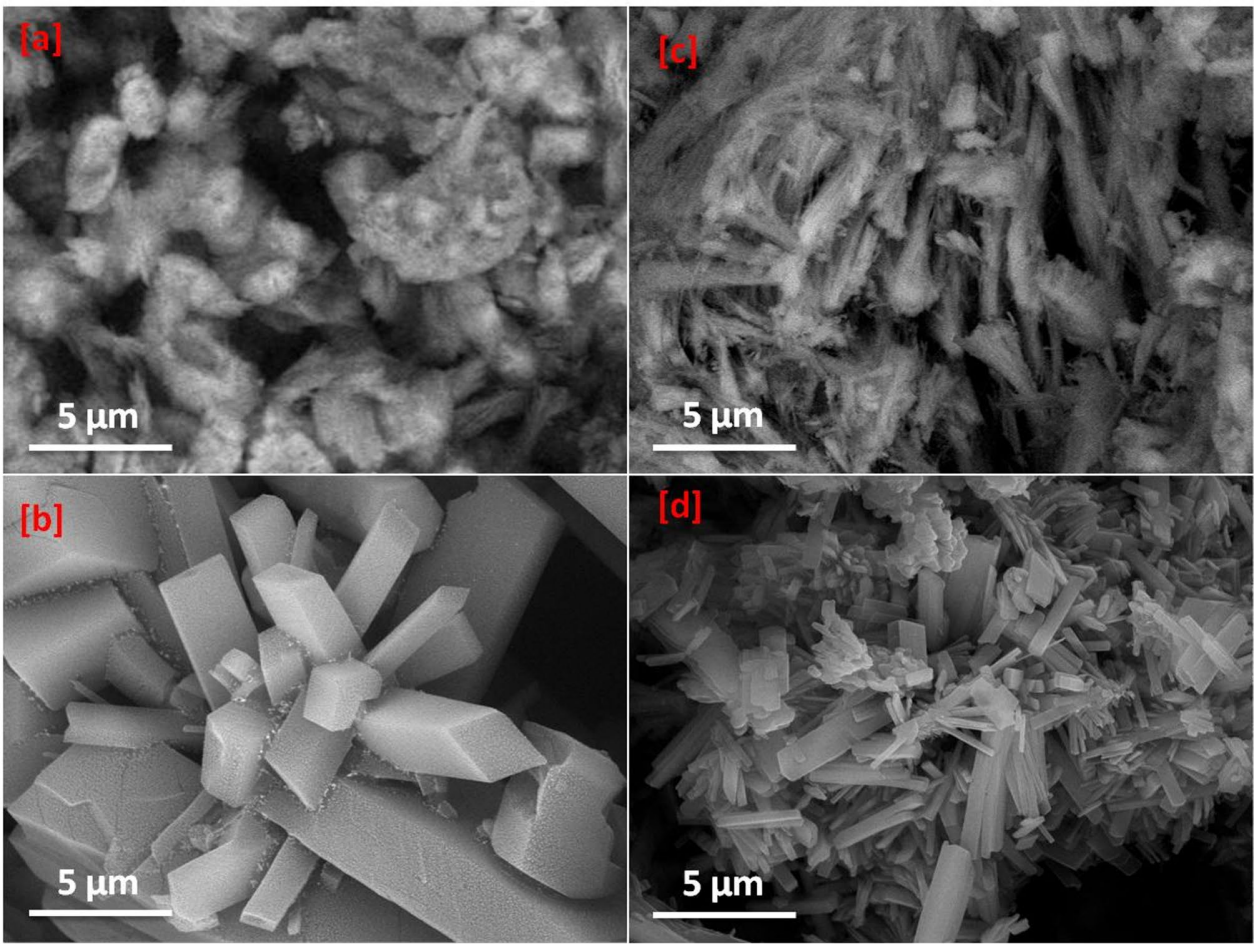
Micrographs; (**a**) ES, (**b**) Ca-BDC (ES), (**c**) FB and (**d**) Ca-BDC (FB).

**Fig. 3:**
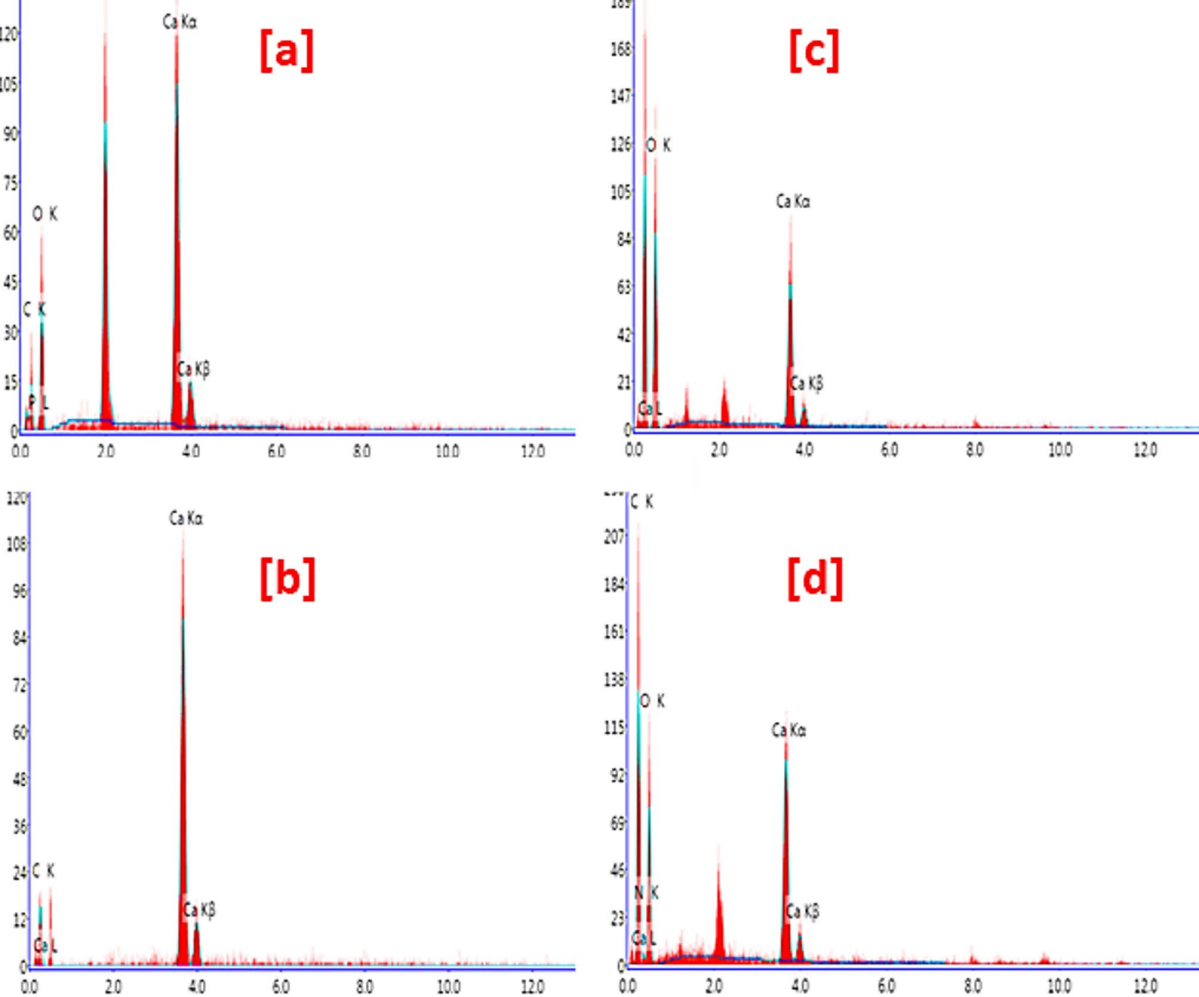
EDX analysis; (**a**) ES, (**b**) Ca-BDC (ES), (**c**) FB and (**d**) Ca-BDC (FB).

Variation in the surface area and reactivity are mainly correlated to the geometrical features that in turn reflected in the adsorption affinity, whereas, the topography of Ca-BDC is dependent on the organic ligands, resource of calcium and the synthetic techniques. Figure 2 represents the scanning electron images for ES & FB compared to Ca-BDC (ES) & Ca-BDC (FB). The micrographs of ES & FB wastes showed that both were exhibited with fibrous structure morphologies (Fig. 2a,c). The obtained Ca-BDC (ES) and Ca-BDC (FB) were shown with orthorhombic crystals (Fig. 2b,d), while smaller crystal size was observed in case of Ca-BDC (FB). The obtained Ca-BDC (ES and FB) has similar morphological and geometrical features with that recently synthesized under solvothermal conditions^64,67,71^. Energy dispersive X-ray (EDX) analysis was presented in Fig. 3 for identification of the elemental composition of the prepared Ca-MOFs. In both Ca-BDC (ES) & Ca-BDC (FB), the characteristic peaks of calcium at 3.8 and 4.0 keV were estimated in addition to the peaks of C at 0.2 keV and O at 0.4 keV. These can inform the chemical composition of the obtained Ca-BDC.

**Fig. 4:**
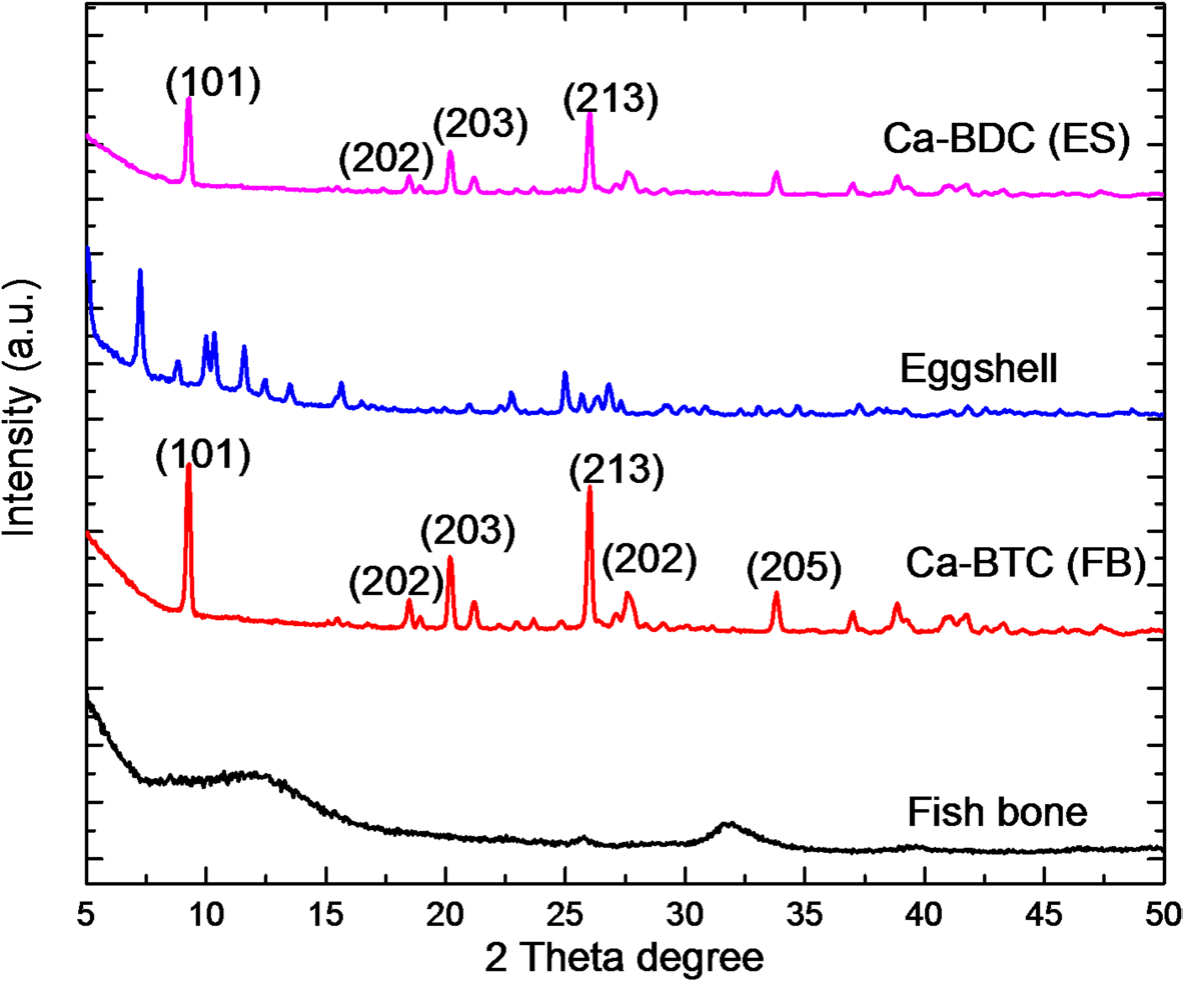
XRD patterns for the synthesized adsorbents of ES, Ca-BDC (ES), FB and Ca-BDC (FB).

X-ray diffractions for the powder samples of ES, Ca-BDC (ES), FB & Ca-BDC (FB) were shown in Fig. 4. It could be declared that, ES waste is more crystalline compared to FB waste. FB waste was shown with two broad peaks at 2*θ* = 12.4° and 31.8°, while FB waste exhibited many signals at 2*θ* = 7.9°, 10.1°, 11.8° and 25.3°. However, both of Ca-BDC (ES) and Ca-BDC (FB) were characterized with six main diffraction peaks at 2*θ* = 8.9°, 18.5°, 20.2°, 26.3°, 27.8° and 34.4°. The recorded diffractions are assigned for (101), (202), (203), (213), (202) and (205) of in orthorhombic crystalline structure of Ca-BDC (JCPDS: 00-047-0703) and these diffractions are in accordance to that previously reported for Ca-MOF^64,67,71–73^. Comparing with Ca-BDC (ES), Ca-BDC (FB) showed more intense diffraction beaks. These data affirmed the successful crystallization of Ca-BDC (ES) & Ca-BDC (FB) from egg shells & fish bones as calcium-based natural wastes under solvothermal conditions.

**Fig. 5:**
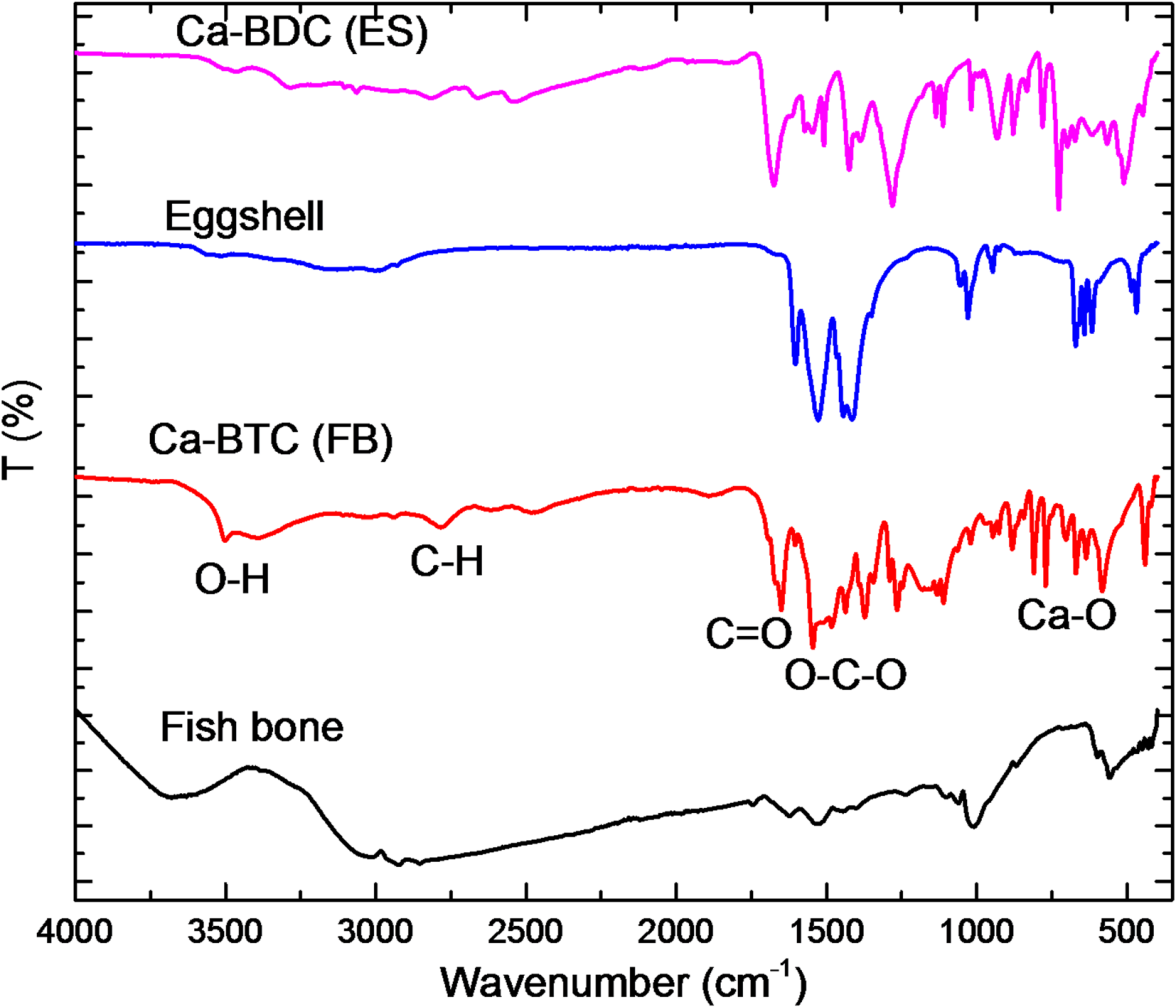
Infrared spectra for the synthesized adsorbents of ES, Ca-BDC (ES), FB and Ca-BDC (FB).

The infrared spectra were presented for the determination of the functional groups of Ca-BDC (ES) & Ca-BDC (FB) compared to ES & FB, for further affirmation of their chemical functionality and accessibility (Fig. 5). As clearly observed, FB spectrum was shown with significant signals at 3700 cm^− 1^, 3050 cm^− 1^, 1600 cm^− 1^, 1550 cm^− 1^, 1000 cm^− 1^ and 600 cm^− 1^, corresponding to O–H stretching (free), O–H stretching (intra-molecular bonding), C=O, C=C, C–O (ester), and C–H bending, respectively. Whereas, ES spectrum was exhibited with number of peaks estimated at 1700 cm^− 1^, 1600 cm^− 1^, 1450 cm^− 1^, 1100 cm^− 1^, 750 cm^− 1^ and 650 cm^− 1^, assigned for C=O, C=C, O–H bending, C–O stretching (ester), C=C bending and C–H bending, respectively^74^. On the other hand, Ca-BDC(FB) was characterized with bands at 1700–1600 cm^− 1^,1450 cm^− 1^, 1250 cm^− 1^, 1100 cm^− 1^, 750 cm^− 1^, 700 cm^− 1^ and 600 cm^− 1^, referring to C=O, C=C, O–H bending, C–O stretching (ester), C=C bending and C–H bending, respectively^75,76^. Whereas, Ca-BDC(ES) was shown with signals at 1750 cm^− 1^, 1600–1650 cm^− 1^, 1400 cm^− 1^, 1250 cm^− 1^ and 950 cm^− 1^, signing to C=O, C=C, O–H bending, C–O stretching (ester) and C=C bending, respectively. Moreover, by comparing between spectra of FB and Ca-BDC(FB), a significant band of M–O (i.e., Ca-O) at 763 cm^− 1^ is clearly observed for Ca-BDC(FB), similarly, by comparing between that of ES and Ca-BDC(ES), the same band is observed at 442 cm^− 1^.This could affirm the successive exploitation of both FB & ES as calcium precursors for preparation of the as-required MOFs via the interaction between the deprotonated carboxylate groups of BDC with Ca ions of both of ES and/or FB, forming the Ca-BDC coordination framework^63,67^.

**Fig. 6:**
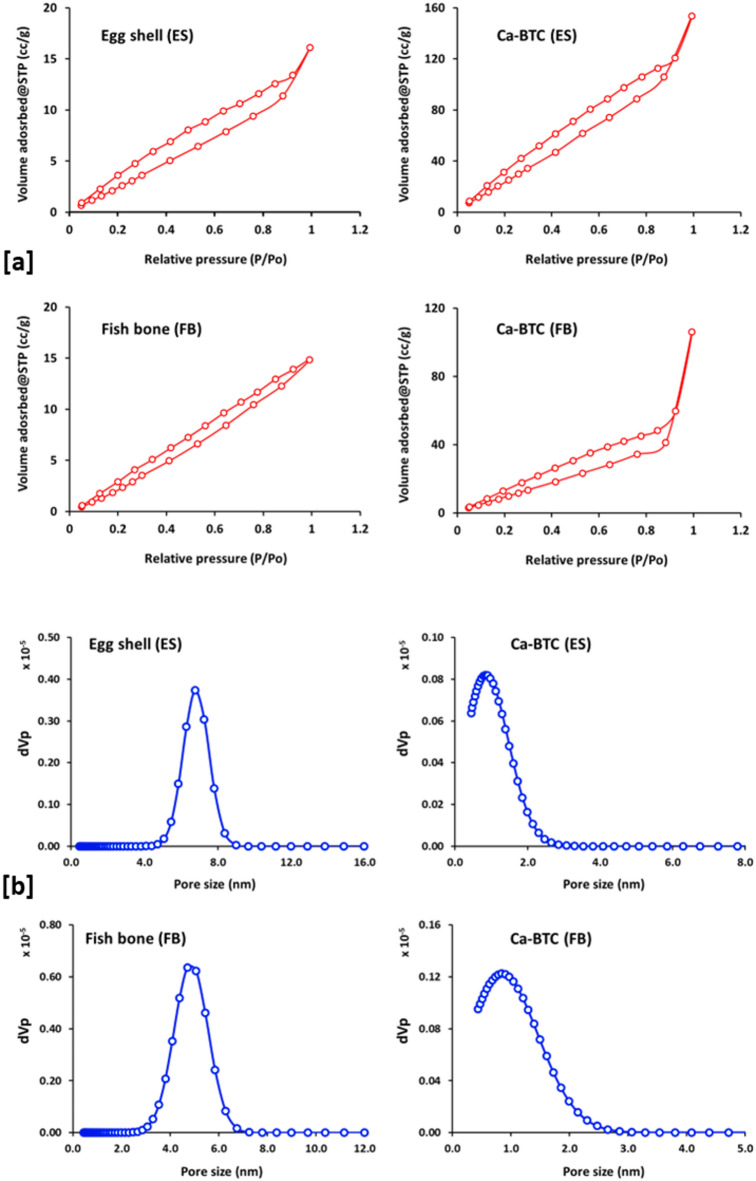
BET isotherm for N_2_ adsorption onto the synthesized adsorbents of ES, Ca-BDC (ES), FB and Ca-BDC (FB).

Figure 6 represents BET isotherm for N_2_ adsorption onto the synthesized adsorbents of ES, Ca-BDC (ES), FB and Ca-BDC (FB). From the plotted data in Fig. 6a, it could be declared that; the volume of adsorbed N_2_ is increased nearly 7 times by exploitation of ES (0.023 cc/g) for preparation of the as-required MOF (0.158 cc/g for Ca-BDC (ES)). Similarly, N_2_ adsorption was increased by rate of 9 times by comparing between FB and Ca-BDC (FB) to be 0.023 cc/g & 0.225 cc/g, respectively. Additionally, the pore size for the ES and FB was ranged in 3.07–8.37 nm which reflect that the ES and FB are classified as mesoporous materials. In case of Ca-BDC (ES) and Ca-BDC (FB), the pore size was reduced to be ranged in 0.44–2.47 nm suggesting the mesoporous character of the prepared Ca-BDC^77,78^. Moreover, Table 1 demonstrates the collected data for surface area to affirm that, the surface area of ES (10.88 m^2^/g) was superiorly widen more than 50 times by its exploitation for preparation of Ca-BDC (ES) (563.16 m^2^/g). Also, in case of FB (27.77 m^2^/g), surface area was increased by rate of 28 times, by its application as calcium precursor for synthesis of Ca-BDC (FB) (721.38 m^2^/g). Whereas, Ca-BDC (FB) (721.38 m^2^/g) exhibited a surface area 1.3 times larger than that of Ca-BDC (ES) (563.16 m^2^/g). These could affirm the effectiveness of both ES & FB for implantation of the as-required highly crystalline/stable/extremely wide surface area BDC to be successively applicable as highly adsorptive materials as currently demanded.

**Table 1:** Surface properties for the synthesized adsorbents.

Adsorbent	Surface area (m^2^/g)	Pore volume (cc/g)	Pore size range (nm)
Egg shell (ES)	10.88	0.023	5.07–8.37
Ca-BDC (ES)	563.16	0.158	0.52–2.46
Fish bone (FB)	27.77	0.032	3.07–6.75
Ca-BDC (FB)	721.38	0.225	0.52–2.64

#### Ammonia removal

The synthesized adsorbents of ES, Ca-BDC (ES), FB and Ca-BDC (FB) were applied in the purification of fish farm samples from ammonia via adsorption. Effect of pH on the ammonia adsorption onto the applied adsorbents [ES, FB, Ca-BDC (ES) and Ca-BDC(FB)] was firstly investigated in order to check the optimal pH for the ammonia removal process. The removal of ammonia was studied at different pH of 4, 5, 6, 7, 8, and 9 as recorded in Figure S1 (supplementary file). The adsorption capacity of ammonia was gradually enlarged from pH 4 (acidic) to pH 7 (neutral) and decreased in alkaline medium (pH = 8, 9). In acidic medium (pH = 4–6) ammonia ions are more stable in solution in the form of NH_4_Cl which cause low removal rate [187.2–206.3 mg/g for Ca-BDC (FB)]^79,80^. While in the alkaline medium (pH = 8–9), the presence of alkali ions in the water is affected on the displacement of ammonia ions and hence reduce the adsorption capacity of ammonia [187.1–276.4 mg/g for Ca-BDC (FB)]^79,80^. The data recorded that, the neutral pH is an optimal medium for ammonia adsorption onto Ca-BDC and the highest adsorption capacity was observed at pH 7.

**Fig. 7:**
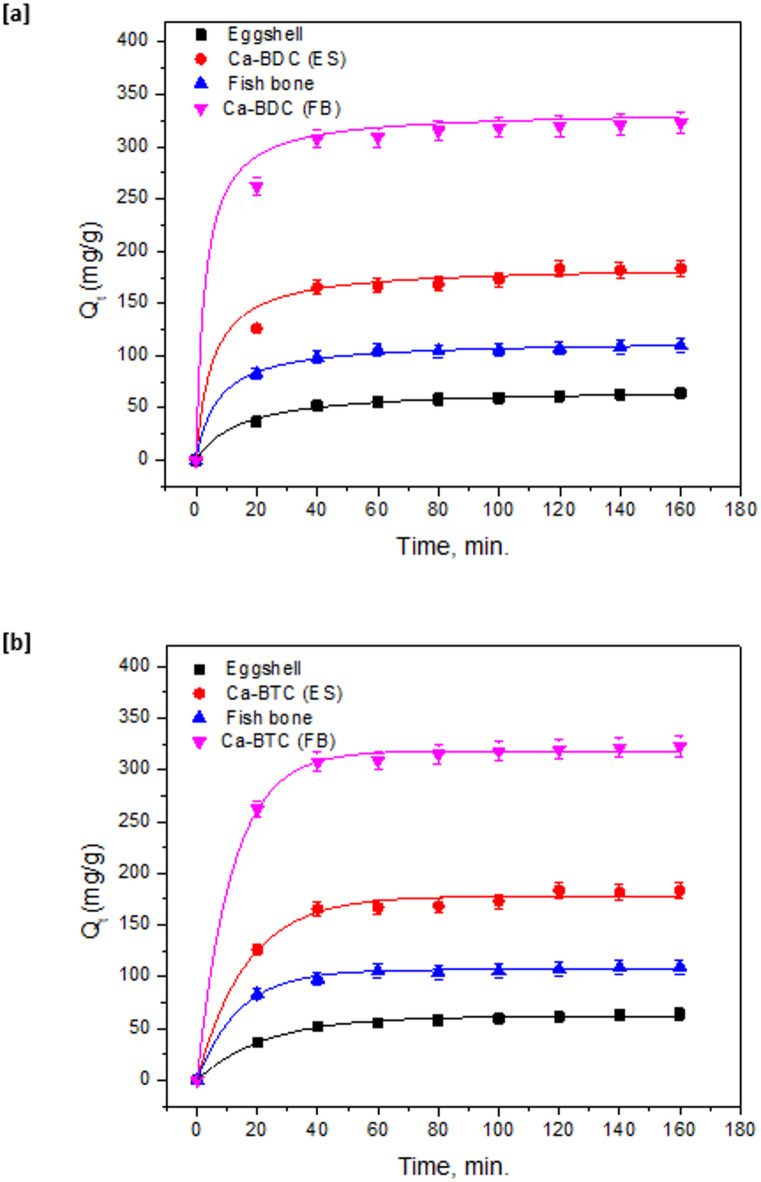
Kinetic for adsorption of ammonia onto the synthesized adsorbents; (**a**) fitting to pseudo-first order and (**b**) fitting to pseudo-second order.

Adsorption of ammonia onto the synthesized BDC was kinetically studied up to 160 min as the data showed in Fig. 7. Figure 7a shows that; the removal of ammonia onto Ca-BDC was observed to be quite fast in the first 40 min and became observably slow. Subsequently, a plateau shape was detected which was attributed to the equilibrium state. The adsorption of ammonia onto Ca-BDC(FB) was significantly higher than that of Ca-BDC(ES). The adsorption amounts of ammonia within the first 40 min were 125 and 270 mg/g onto Ca-BDC(ES) & Ca-BDC(FB), respectively. The adsorption contents after 160 min reached 180 and 320 mg/g for ammonia, respectively. From the adsorption results, the removal amounts onto Ca-BDC(FB) were higher than that of Ca-BDC(ES) to be nearly doubled. From the nonlinear fitting of adsorption data for the pseudo-first ordered (Fig. 7a) and pseudo-second ordered (Fig. 7b) reaction, the kinetic parameters were evaluated and inserted in Table 2. The estimated key factors are adsorption capacity (Qe, mg/g), rate constant (K_1_, min^− 1^ & K_2_, L/mg min), determination coefficient (R^2^) and the Chi square test (x^2^) which were evaluated to be recognized as the fitting analysis parameters. According to the linearity in the plotted figures and the estimated R^2^ and x^2^ values, the ammonia adsorption is more fitted to the pseudo-second ordered reaction rather than the pseudo-first ordered modeling. Fitting to the pseudo-second ordered reaction reflects that the ammonia adsorption onto the applied BDC was correlated to the concentration of ammonia and the dosage of Ca-BDC^81,82^. This concept assumes that the adsorption capacity of ammonia could be further improved via the increment of BDC dosage. The estimated data of adsorption capacity of ammonia are 68.86 mg/g for ES and 185.91 mg/g for Ca-BDC (ES). While, the calculated adsorption capacity was enlarged from 114.53 mg/g for FB to 334.17 mg/g for Ca-BDC (FB). The estimated data show that, the ammonia adsorption capacity was increased by factor of 1.2–1.3 by using Ca-BDC (FB) instead of Ca-BDC (ES). The rate constant of K_2_ was increased from 1.10 × 10^− 3^ L/mg min and 1.33 × 10^− 3^ L/mg min to 1.22 × 10^− 3^ and 1.44 × 10^− 3^ L/mg min for Ca-BDC (ES) and Ca-BDC (FB).

**Table 2:** Parameters of adsorption kinetic of ammonia onto the synthesized adsorbents.

Adsorbent	Pseudo-first order				Pseudo-second order			
	Q_e_ (mg/g)	K_1_ × 10^− 3^ (min^− 1^)	*R* ^2^	X^2^	Q_e_ (mg/g)	K_2_ × 10^− 3^ (L/mg min)	*R* ^2^	X^2^
Egg shell	61.77	44.04	0.96	27.12	68.86	1.10	0.99	1.82
Ca-BDC (ES)	178.00	60.84	0.95	31.51	185.91	1.22	0.98	7.63
Fish bone	106.95	72.03	0.97	38.32	114.53	1.33	0.99	2.47
Ca-BDC (FB)	318.17	86.10	0.96	13.32	334.17	1.44	0.99	1.56

**Fig. 8:**
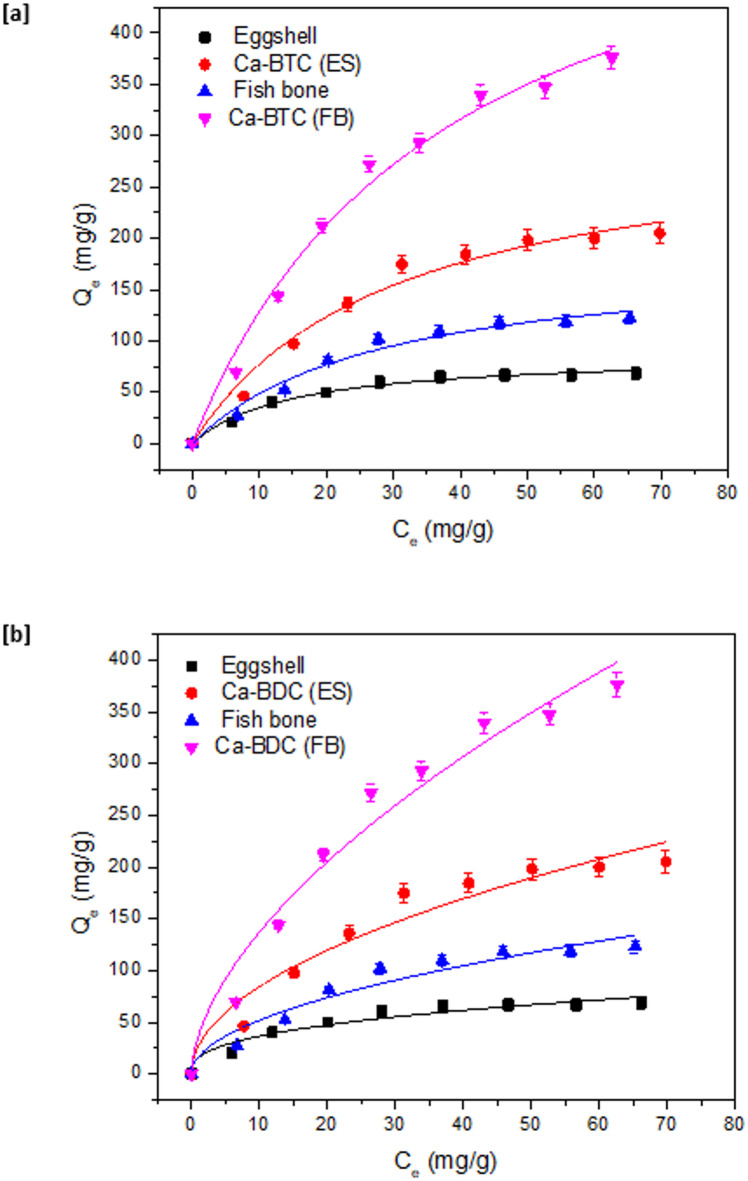
Adsorption isotherm of ammonia onto the synthesized adsorbents; (**a**) fitting to Langmuir model and (**b**) fitting to Freundlich model.

The effect of ammonia concentration in the wastewater sample of fish farm was studied as demonstrated in Fig. 8. At low concentration (less than 45 mg/g), the removal amounts of ammonia by any of the examined adsorbents was high. While at higher concentration (higher than 45 mg/g), adsorption saturation is notified and the increment in the adsorption capacity was quite low. The adsorption capacity data of ammonia is represented with nonlinear fitting for the Langmuir (Fig. 8a) and Freundlich isotherm (Fig. 8b). Langmuir and Freundlich isotherms are two adsorption key parameters that are largely applied for the explanation of adsorption results. Freundlich isotherm is used for the multi-layered adsorption in the heterogeneous materials, while the Langmuir model is exploited for the mono-layered adsorption onto the homogeneous materials. The key factors of isotherm as adsorption maximum capacity (Q_m_, mg/g), Freundlich constant (n), rate constant of isotherm (K_L_, K_F_), determination coefficient (R^2^) and Chi square test (x^2^) were estimated and represented in Table 3. Additionally, Temkin and Dubinin isotherm models are also measured for fitting the adsorption data of ammonia (Supplementary file, Figure S1) and their different parameters such as K_T_, b, q_s_, E and R^2^ are all calculated and inserted in Table 3. From the demonstrated fitting data and the evaluated parameters, the Langmuir model shows more fitting for the adsorption isotherm of ammonia, while higher R^2^ values and less x^2^ values were reported in case of Langmuir isotherm comparing with Freundlich isotherm. The evaluated maximum adsorption capacities (Q_m_) onto ES & Ca-BDC (ES) were 86.99 mg/g and 308.16 mg/g, respectively. While higher maximum capacities onto FB & Ca-BDC (FB) were obtained with values of 184.28 mg/g and 616.11 mg/g, respectively. The maximum adsorption capacity of ammonia observed to be duplicated when Ca-BDC (FB) applied comparing with Ca-BDC (ES). Furthermore, good linearity was observed for Temkin model (as seen in Figure S1, supplementary file) and high regression coefficient (R^2^ = 0.97–0.99, Table 3), while Dubinin model didn’t show good fitting. Model of Temkin explains that the adsorption of ammonia is characterized by the uniform distribution of the energies of binding, until the maximum binding energy^83^ Therefore, Temkin model supports the Langmuir isotherm fitting with the uniform distribution, while the Langmuir model more useful due to estimation of maximum capacity. Form the Dubinin model, the estimated free energy (E) of ammonia adsorption was ranged in 11.30–28.62 kJ/M which reflecting that the physical sorption (free energy = 0–40 kJ/M) played the main role in ammonia adsorption^84–86^. The physical sorption describes weak interactions including Vander Waals, hydrophobic bonding, dipole-dipole interactions, coordination bond and hydrogen bond^84–86^. Based on the as-illustrated data, it can be summarized that; the optimal conditions for ammonia removal (50 mg/L) is identified as follows; at 25 °C, 0.5 g of Ca-BDC (dose), adsorption duration of 40 min and pH 7.

**Table 3:** Parameters of adsorption isotherm of ammonia onto the synthesized adsorbents.

Isotherm	Parameter	Egg shell	Ca-BDC (ES)	Fish bone	Ca-BDC (FB)
Langmuir	Q_m_ (mg/g)	86.99	308.16	184.28	616.11
	K_L_ × 10^− 3^ (mg/L)	67.94	33.44	35.76	26.33
	R^2^	0.99	0.98	0.98	0.99
	X^2^	7.08	119.11	49.41	188.09
Freundlich	n	2.66	1.99	1.98	1.72
	K_F_	15.36	26.56	16.27	36.03
	R^2^	0.95	0.94	0.94	0.96
	X^2^	29.86	308.88	119.95	567.82
Temkin	K_T_ (L/M)	1.77	3.89	3.56	4.15
	b × 10^− 3^ (J g/M^2^)	21.50	5.75	9.69	3.10
	R^2^	0.97	0.99	0.98	0.99
Dubinin	q_s_ (M/g)	67.88	209.11	126.69	359.91
	E (kJ/M)	11.30	17.52	18.05	28.62
	R^2^	0.92	0.96	0.96	0.95

From the summarized studies (Table 4), the maximum adsorption capacity of ammonia onto the activated carbons and biochar derived from different sources^79,87–91^ was significantly smaller (5.4–146.0 mg/g) compared to that obtained herein for Ca-BDC (FB) (616.11 mg/g). Also, very low maximum removal of ammonia as ammonium chloride (5.05–17.70 mg/g) was recorded by different zeolites from different natural sources (zeolite, clinoptilolite, mordenite)^92–98^. Different MOF materials (including, MIL-53, MIL-53-NH_2_, MIL-100, MIL-101, MOF-74, MOF-76, ZIF-8, UiO-67) were recently employed in adsorption of ammonia gas which can’t be compared with the adsorption results here for ammonium chloride^56–61^. While using of MOF of UiO-67-ox-Cu showed ammonia adsorption with maximum capacity of 178.3 mg/g^99^ which is considerably lower than the obtained for Ca-BDC (FB) by 3.4 times.

**Table 4:** Comparison the removal capcity of ammonia by different adsorbent based on previous studies.

Adsorbent	Maximum adsorption capacity (mg/g)	References
Ca-BDC	616.11	Present study
Biochar	8.60–146.00	^79,87–91^
Zeolite	5.05–11.31	^94,95,98^
Clinoptilolite	6.59–17.70	^92–94,96^
Mordenite	8.70–15.13	^95,97^
UiO-67-ox-Cu	178.30	^99^

**Fig. 9:**
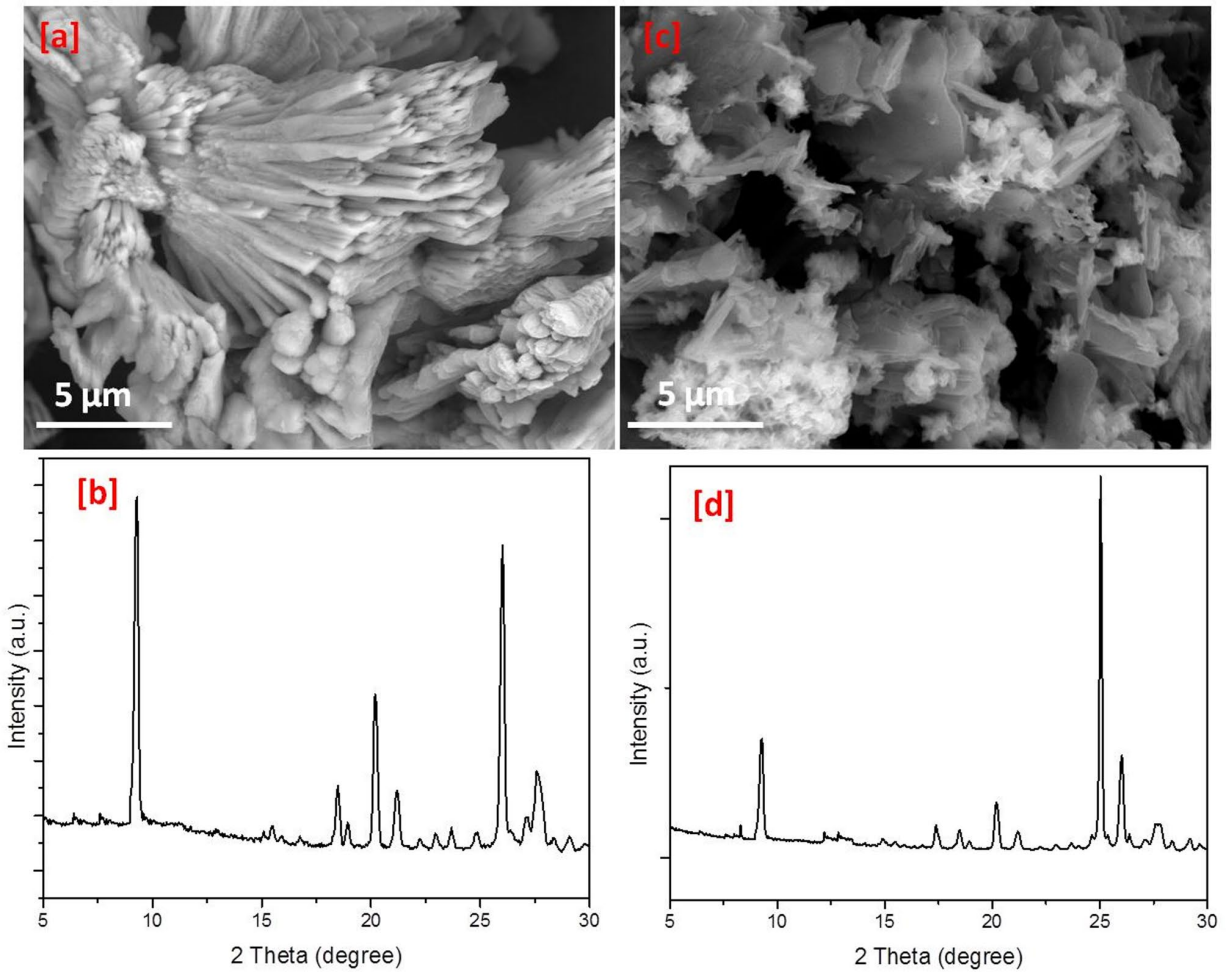
Micrographs and XRD after ammonia adsorption (**a**,**b**) Ca-BDC (ES), (**c**,**d**) Ca-BDC (FB), (**a**,**c**) Micrographs and (**b**,**d**) XRD patterns.

The microscopic observation and the geometrical features for the Ca-BDC after ammonia adsorption were examined and the data represented in Fig. 9a,c. The fibrous structures for the applied Ca-BDC (ES and FB) was not significantly changed after ammonia adsorption and some defects in the orthorhombic crystal were obtained relating to the ammonia adsorption process. Additionally, the XRD Fig. 9b,d was measured for Ca-BDC after ammonia adsorption to further investigate its stability. The essential diffraction peaks at 2*θ* = 8.9° (101), 18.5° (202), 20.2° (203), 26.3° (213) and 27.8° (202) were clearly appeared for both of Ca-BDC (ES) and Ca-BDC (FB) after adsorption of ammonia, which confirmed that the crystalline structure of Ca-BDC (ES and FB) was not affected by adsorption process. Both of microscopic observations and XRD data for the used Ca-BDC (ES and FB) revealed that the geometrical features and the crystalline structure for Ca-BDC was not considerably affected by ammonia adsorption and reflected stability Ca-BDC towards application in ammonia removal which supports its practical applications in ammonia removal rather than the poor stability MOFs.

**Fig. 10:**
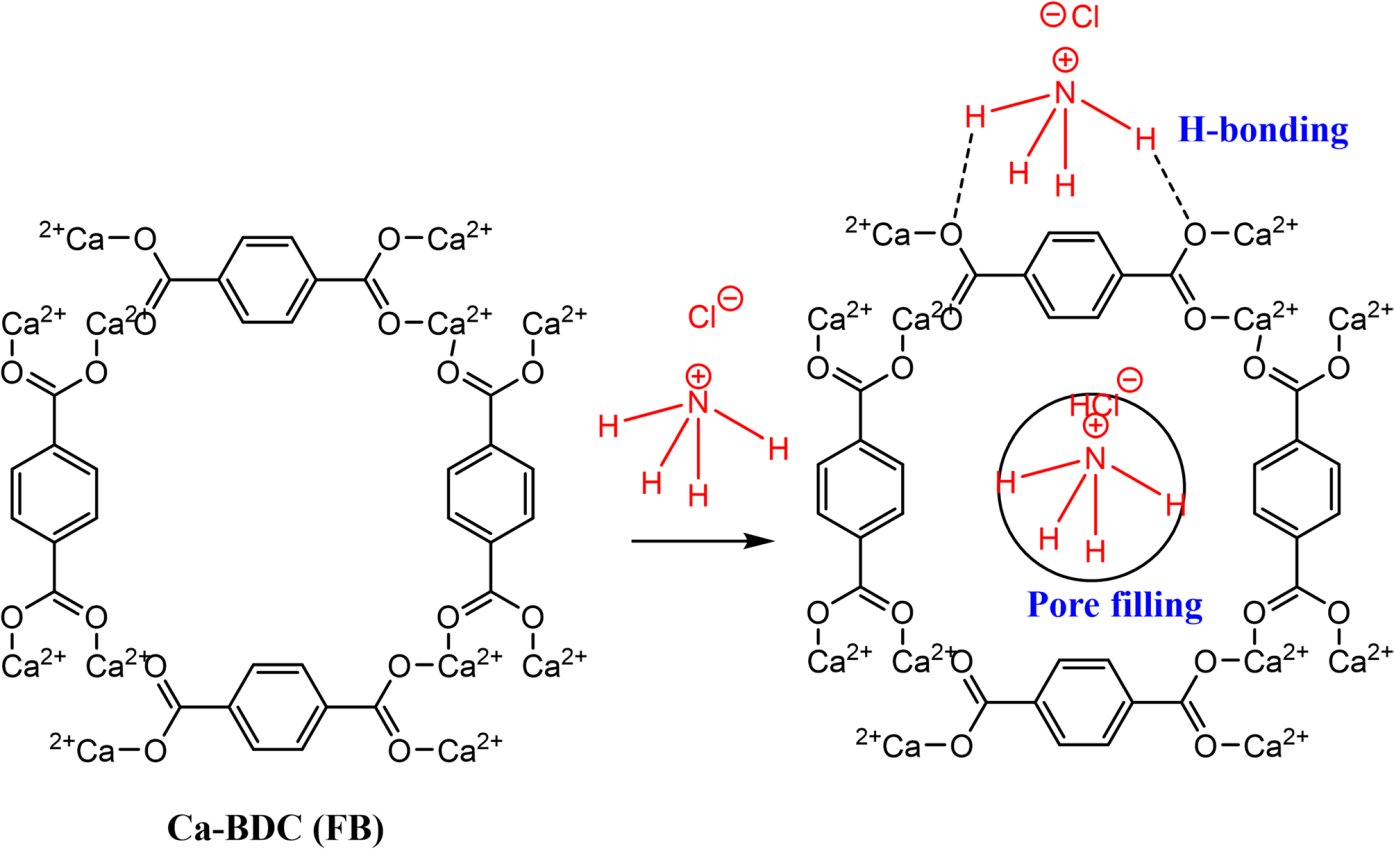
Adsorption mechanism of ammonia onto Ca-BDC.

Adsorption of ammonia with the synthesized Ca-BDC could be hypothesized to be proceeded via the physical and chemical adsorption as suggested in Fig. 10. Ammonia could be physically inter-trapped within the pores of Ca-BDC which supported the physical sorption process^84,100,101^. Moreover, the adsorption of ammonia may be occurred via weak chemical interaction with Ca-BDC. Also, the Van der Waals forces, dipole-dipole interaction, hydrogen bond and coordination bonding may be formed, attributing to the free energy values. Hydrogen bonding between the oxygen of carboxylic group in Ca-BDC and hydrogen of ammonia and coordination bonding between Ca of Ca-BDC and nitrogen in ammonia could occur^3,30^. Moreover, n–π interaction is suggested to be observed between the benzene rings in Ca-BDC and lone pair of electrons in ammonia^102,103^. The adsorption capacity of ammonia onto Ca-BDC (FB) was quite larger than Ca-BDC (ES), which could be explained by the higher surface area of Ca-BDC (FB) and subsequently increase the adsorption capacity. The fitting to Langmuir isotherm informed that the adsorption of ammonia is limited by the functional groups/pores of Ca-BDC and therefore a plateau shape is observed in the plotted data. Meaning that, the mono-layered adsorption is principally obtained via the adsorption of ammonia at each binding site of Ca-BDC and characterized by the uniform distribution according to the Temkin model^84,104,105^.

#### Regeneration study

**Fig. 11:**
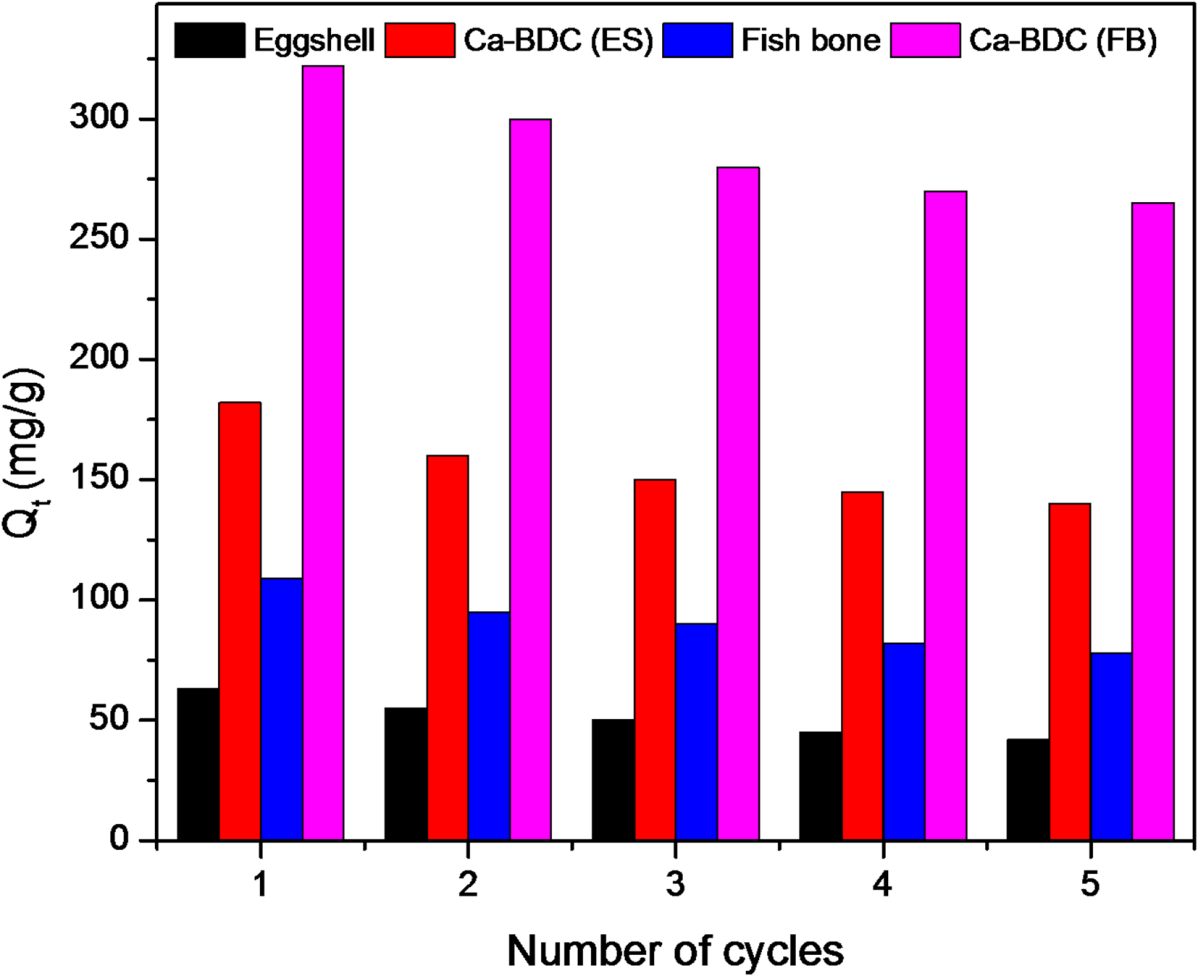
Effect of the repetitive recycles on the adsorption of ammonia onto Ca-BDC.

As formerly reported (Fig. 9), the applied Ca-BDC showed good stability in morphological and crystalline properties which support its reusability. Furthermore, the regeneration of the applied Ca-BDC towards the removal of ammonia was studied up to 5 regenerative cycles, while each cycle contains adsorption and desorption step. At the end of the first adsorption step, the applied Ca-BDC was washed by ethanol followed by water to remove the adsorbed ammonia (as desorption step), dried at room temperature and then applied in the next adsorption step. The regeneration process was repeated to apply five cycles and the obtained results presented in Fig. 11. The determined data showed that the adsorption of ammonia onto Ca-BDC was progressively decreased by the regeneration process, which could be related to the leaching of some Ca-BDC to during the regeneration step. After five regeneration cycles, the removal capacity of ammonia is lowered from 185.91 mg/g and 334.17 mg/g to 140.12 mg/g and 265.60 mg/g in case of Ca-BDC (ES) and Ca-BDC (FB), respectively. The decrement in adsorption capacity of ammonia was 24.6% for Ca-BDC (ES) and 20.5% for Ca-BDC (FB). The data reflect the effective regeneration of Ca-BDC and the applied Ca-BDC (FB) exhibited quite good removal capacity of ammonia which suggest the wide-scale applicability of the synthesized Ca-BDC (FB) from biogenic waste in the ammonia removal or pollutants adsorption in general.

## Conclusion

Herein, the current approach represents a comparable study between fish bones (FB) and egg shells (ES) as biogenic wastes to act as calcium precursors for synthesis of Ca-BDC via solvo-thermal technique, abbreviated as Ca-BDC(FB) & Ca-BDC(ES), respectively. The adsorption kinetic key factors, adsorption isotherm and regeneration were also studied. The adsorption of ammonia followed the pseudo-second ordered reaction and Langmuir isotherm. FB showed to be more preferable rather than ES as calcium precursor for preparation of highly efficient Ca-BDC, attributing to smaller size of Ca-BDC (FB) rather than that of Ca-BDC (ES) is reflected in larger surface area and in turn higher absorptivity for more efficient removal of ammonia. The evaluated maximum adsorption capacities (Q_m_) onto ES & Ca-BDC (ES) were 86.99 mg/g and 308.16 mg/g, respectively. The maximum capacities of ammonia onto FB & Ca-BDC (FB) were 184.28 mg/g and 616.11 mg/g, respectively. Whereas, Ca-BDC (FB) (721.38 m^2^/g) was shown with significantly wider surface area by factor of 1.3 by comparing with Ca-BDC (ES) (563.16 m^2^/g). The synthesized Ca-BDC showed quite good regenerative and the adsorption capacity of ammonia was lowered by 24.6% for Ca-BDC (ES) and 20.5% for Ca-BDC (FB). Overall, the study provides a promising future trend for in designing MOFs with appropriate central metals using different bio-wastes with the principal of waste treat waste, to be sequentially applicable for capturing of NH_3_.

## Electronic supplementary material

Below is the link to the electronic supplementary material.

Supplementary Material 1

## Data Availability

The datasets used and/or analysed during the current study available from the corresponding author on reasonable request.
