# Supplementary material for: Ammonia removal from simulated fish farms by metal organic framework ingrained by egg shell and fish bones
Source: Sci Rep. 2025 May 16;15:17086. doi: 10.1038/s41598-025-01827-0 (PMC12084629; doi:10.1038/s41598-025-01827-0)
Supplement: Supplementary file 1 — Supplementary Material 1 [file 41598_2025_1827_MOESM1_ESM.docx]

**Supporting information**

**Table S1:** Elemental analysis of prepared MOFs

| Sample | Molecular formula | Ca^2+^ * | C ** | H |
| --- | --- | --- | --- | --- |
| Ca-BDC (ES) | Ca(DDC)(H_2_O) | 18.04± 0.51 | 43.31  (43.24) | 2.81  (2.72) |
| Ca-BDC (FB) | Ca(DDC)(H_2_O)_2_ | 16.68± 0.74 | 40.10  (40.00) | 3.38  (3.36) |

*Data obtained from ICP analysis, ** the calculated data between brackets and the obtained data from CHN analysis

**Figure S1:** Effect of pH on the removal of ammonia by the synthesized adsorbents.

**Figure S2:** Adsorption isotherm of ammonia onto the synthesized adsorbents; **[a]** Temkin model and **[b]** Dubinin model.

| **[a]** |  |
| --- | --- |
| **[b]** |  |
